# Epcoritamab induces potent anti-tumor activity against malignant B-cells from patients with DLBCL, FL and MCL, irrespective of prior CD20 monoclonal antibody treatment

**DOI:** 10.1038/s41408-021-00430-6

**Published:** 2021-02-18

**Authors:** Hilma J. van der Horst, A. Vera de Jonge, Ida H. Hiemstra, Anne T. Gelderloos, Daniella R. A. I. Berry, Nathalie J. Hijmering, Hendrik F. van Essen, Daphne de Jong, Martine E. D. Chamuleau, Sonja Zweegman, Esther C. W. Breij, Margaretha G. M. Roemer, Tuna Mutis

**Affiliations:** 1 Department of Hematology, Cancer Center Amsterdam, Amsterdam University Medical Center, Location VUMC, Amsterdam, Netherlands; 2 Genmab, Utrecht, Netherlands; 3 Department of Pathology, Amsterdam University Medical Center, Location VUMC, Amsterdam, Netherlands

**Keywords:** Immunotherapy, Cancer immunotherapy, B-cell lymphoma

## Abstract

Epcoritamab (DuoBody-CD3xCD20, GEN3013) is a novel bispecific IgG1 antibody redirecting T-cells toward CD20^+^ tumor cells. Here, we assessed the preclinical efficacy of epcoritamab against primary tumor cells present in the lymph node biopsies from newly diagnosed (ND) and relapsed/refractory (RR) B-NHL patients. In the presence of T-cells from a healthy donor, epcoritamab demonstrated potent activity against primary tumor cells, irrespective of prior treatments, including CD20 mAbs. Median lysis of 65, 74, and 84% were achieved in diffuse large B-cell lymphoma (*n* = 16), follicular lymphoma (*n* = 15), and mantle cell lymphoma (*n* = 8), respectively. Furthermore, in this allogeneic setting, we discovered that the capacity of B-cell tumors to activate T-cells was heterogeneous and showed an inverse association with their surface expression levels of the immune checkpoint molecule Herpesvirus Entry Mediator (HVEM). In the autologous setting, when lymph node (LN)-residing T-cells were the only source of effector cells, the epcoritamab-dependent cytotoxicity strongly correlated with local effector cell-to-target cell ratios. Further analyses revealed that LN-residing-derived or peripheral blood-derived T-cells of B-NHL patients, as well as heathy donor T-cells equally mediated epcoritamab-dependent cytotoxicity. These results show the promise of epcoritamab for treatment of newly-diagnosed or relapsed/refractory B-NHL patients, including those who became refractory to previous CD20-directed therapies.

## Introduction

The B-cell specific marker CD20, which is expressed on all mature B-cells, but absent on hematopoietic stem cells, pro-B-cells and plasma cells^1^, is an attractive target for the treatment of B-cell malignancies, including B-cell non-Hodgkin lymphomas (B-NHL). Consequently, over the past decades, several CD20-targeting monoclonal antibodies (mAbs), including rituximab and subsequent-generation CD20-mAbs, have been successfully applied for the treatment of B-NHL, often in combination with chemotherapy^2^. Nonetheless, a high incidence of disease relapse occurs after treatment with currently available CD20-targeting mAbs, urging the development of more innovative and powerful therapies targeting CD20. While conventional mAbs can eliminate target cells via several mechanisms, they do not exploit the powerful cytotoxic machinery of T-cells, which can induce long-term remissions in several hematopoietic cancers and solid tumors, provided that they are efficiently and specifically targeted to tumor cells^3,4^. Hence, major efforts are devoted to developing efficient chimeric antigen receptors (CARs) and T-cell engagers targeting CD20^5–11^. Epcoritamab (DuoBody®-CD3xCD20, GEN3013) is a novel full-length IgG1 bispecific antibody (bsAb) redirecting CD3^+^ T-cells to CD20 expressing cells^7,12,13^. In an initial study, we have shown that epcoritamab induces potent T-cell-mediated cytotoxicity towards B-cell NHL cell lines in vitro and in vivo^7^. In the dose-escalation part of an ongoing first-in-human clinical trial, epcoritamab showed a favorable safety profile and preliminary efficacy data indicate encouraging antitumor activity as a single agent, including complete responses, in patients with R/R DLBCL or FL^14^.

Here, we investigated the preclinical activity of epcoritamab using primary tumor cells obtained from lymph node (LN) or bone marrow (BM) biopsies samples of patients with diffuse large B-cell lymphoma (DLBCL; *n* = 16), follicular lymphoma (FL; *n* = 15) and mantle cell lymphoma (MCL; *n* = 8). The patients were newly diagnosed (ND) or relapsed/refractory (RR) to conventional treatment regimens, including anti-CD20 mAbs. We investigated (i) the intrinsic sensitivity of tumor cells to epcoritamab-dependent T-cell-mediated cytotoxicity and the capacity of tumor cells to activate T-cells, ii) whether type of malignancy and prior exposure to CD20-targeted therapy influenced their sensitivity, and (iii) the intrinsic capacity of tumor-associated T-cells to mediate epcoritamab-dependent cytotoxicity.

## Materials and methods

### Cell lines

B-NHL cell lines (Daudi; ATCC and WSU-DLCL2; DSMZ) were cultured in RPMI-1640 (Invitrogen, Carlsbad, CA, USA), supplemented with 10% fetal bovine serum (Invitrogen) and 1% penicillin-streptomycin (Life Technologies, Carlsbad, CA, USA). Cell lines were authenticated by STR-profiling and tested for mycoplasma contamination.

### Patient samples

Single cell suspensions from LN biopsies were isolated by mechanical disruption as described in online supplementary material and methods. Mononuclear cells of BM (BMMCs) or PB (PBMCs) were isolated by Ficoll density-gradient centrifugation. Cells were either used directly or cryopreserved in liquid nitrogen until further use. This type of study does not require approval from an ethics committee. All patient material and clinical data were collected after obtaining informed consent and according to the *code of conduct for medical research* developed by The Council of the Federation of Medical Scientific Societies (FEDERA).

### Phenotyping by flow cytometry

Multicolor flow cytometry was used to identify CD19^+^ and/or CD20^+^ B-cells, CD3^+^CD4^+^ and CD3^+^CD8^+^ conventional T-cells and CD3^+^CD4^+^CD25^+^CD127^low/−^ regulatory T-cells. Malignant B-NHL cells were identified within the total B-cell population on light chain restriction and on tumor-specific combinations with CD markers such as CD5 and CD10 when relevant. When indicated, CD20 expression was quantified on malignant B-cells using an indirect immunofluorescence assay (QIFIKIT®, Agilent Technologies, Santa Clara, CA, USA). Additionally, cell suspensions were phenotyped for immune checkpoint ligands and receptors, including programmed death-ligand 1 (PD-L1), HLA-DR, herpesvirus entry mediator (HVEM), programmed cell death protein 1 (PD-1), T-cell immunoglobulin and mucin domain-containing protein 3 (TIM-3), lymphocyte-activation gene 3 (LAG-3) and B-lymphocyte and T-lymphocyte attenuator (BTLA). Further details are provided in online supplementary material and methods, including an overview of all antibodies used for these studies (Supplementary Table 1).

### Cytotoxicity assays

Violet tracer (Thermo Fisher, Waltham, MA, USA) labeled target cells were pre-incubated with serial dilutions of antibodies for 15 min in 96-well U bottom plates, and then cultured for 24 h without additional effector cells or with effector cells at a fixed (10:1) T-cell to target (E:T) cell ratio. Effector cells were allogeneic or autologous effector PBMCs or, from specified samples, CD3^+^ lymph node-residing T-cells, isolated using magnetic-activated-cell sorting (MACS) following the manufacturer’s instructions (Miltenyi Biotec, Bergisch Gladbach, Germany). Viable target cells were identified with multicolor flow cytometry. Data were analyzed using FACS DIVA software. Cytotoxicity was calculated only if >500 viable target cells were counted in untreated wells and with the following formula:

$${\mathrm{\% }}\;{\rm{Cytotoxicity}} = (100 - {\mathrm{\% }}\;{\rm{viability}}),$$

$${\mathrm{\% }}\;{\rm{Viability}} = 100 \ast \left( {\frac{{\# 7{\rm{AAD}}\_{\rm{negative}}\;{\rm{events}}\;{\rm{of}}\;{\rm{test}}\;{\rm{sample}}}}{{\# 7{\rm{AAD}}\_{\rm{negative}}\;{\rm{events}}\;{\rm{of}}\;{\rm{control}}\;{\rm{sample}}}}} \right).$$

Further details are described in online supplementary material and methods.

The concentration of granzyme B released in cell-free supernatants of cytotoxicity assays was measured by a sandwich ELISA kit (Mabtech, Nacka Strand, Sweden) following manufacturer’s protocol.

### Multiplexed immunofluorescence (mIF)

Multiplexed immunofluorescence was performed on 4 µm-thick formalin-fixed, paraffin-embedded whole tissue sections using the Opal 7-color fluorescence immunohistochemistry kit (Akoya biosciences, Menlo Park, CA, USA). Further details are provided in online supplementary material and methods, including an overview of antibodies used for mIF (Supplementary Table 2).

### Statistics

Dose-response curves were generated and half maximal effective concentration (EC_50_) values calculated using non-linear regression analysis (sigmoidal dose-response) (Graphpad Prism 8.2). Comparisons between variables were performed using Mann–Whitney *U*-test, Wilcoxon matched-pairs signed rank test or unpaired *t*-test for analysis of two groups, and Kruskal–Wallis with Dunn’s multiple comparions test for analysis of multiple groups. Two-sided *p* values <0.05 were considered statistically significant.

## Results

### Epcoritamab induces potent cytotoxicity in the presence of healthy donor PBMCs

**Fig. 1:**
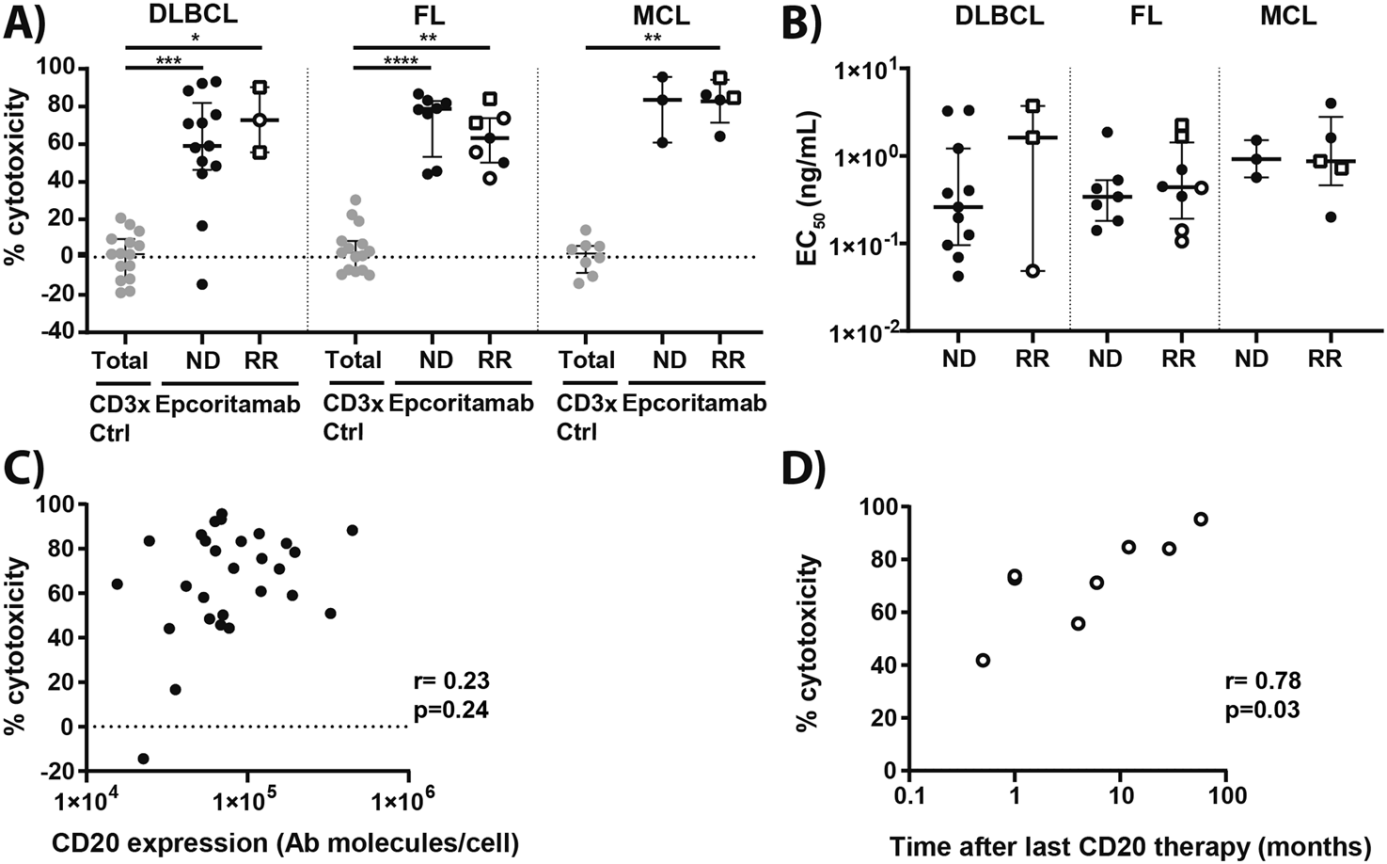
Epcoritamab mediates comparable levels of cytotoxicity in various B-NHL subtypes and in ND and RR samples, including samples from patients who received prior CD20-antibody containing treatment. **A** Percentages cytotoxicity mediated by epcoritamab and CD3xCtrl (30 ng/mL) and **B** EC_50_ values (ng/mL), shown for samples with clear dose-response curves, for cytotoxicity in the presence of allogeneic PBMCs (E:T ratio 10:1) in ND and RR DLBCL (*n* = 16), FL (*n* = 15), and MCL (*n* = 8) samples (median ± interquartile range), including patients who relapsed from or were refractory to CD20 therapy ( and , respectively). Statistical analysis was performed with Kruskal–Wallis and Dunn’s multiple comparisons test to compare epcoritamab with CD3xCtrl for each indication (*p < 0.05; **p < 0.01; ***p < 0.001; ****p < 0.0001) and epcoritamab-dependent cytotoxicity between subtypes (ns (**C**) CD20 expression (antibody molecules per cell) on tumor cells in samples from B-NHL patients unexposed to prior CD20 therapy correlated to epcoritamab-dependent cytotoxicity (30 ng/mL) (Spearman’s; ns). **D** Time between last CD20-therapy and moment of lymph node biopsy (months) of patients previously treated with CD20-antibody containing treatment (*n* = 8) correlated to cytotoxicity mediated by epcoritamab (30 ng/mL) (Spearman’s; *r* = 0.78, *p = 0.03).

To determine the sensitivity of B-NHL cells to epcoritamab, independent of interpatient variation in effector T-cell frequency or function, malignant cells obtained from B-NHL patients were exposed to epcoritamab in the presence of PBMCs of a single healthy donor as a source of effector cells at a fixed 10:1 effector (T-cell) to target (malignant cell) ratio. At a concentration of 30 ng/mL, epcoritamab mediated effective and comparable levels of cytotoxicity in DLBCL (median 65%; range 0–93%; *n* = 16), FL (median 69%; range 42–87%; *n* = 15), and MCL (median 84%; range 61–96%; *n* = 8) samples. In 37/39 samples the EC_50_ values could be reliably calculated and ranged between 0.04–4.0 ng/mL with no significant differences between DLBCL, FL, and MCL in this cohort (median; range: 0.32; 0.04–3.7 ng/mL in DLBCL, 0.42; 0.11–2.1 ng/mL in FL, and 0.89; 0.20–4.0 ng/mL in MCL) (Fig. 1A, B). Epcoritamab was effective in samples from ND patients (median 73%; range 0–96%; *n* = 24) as well as in samples from RR patients (median 73%; range 42–95%; *n* = 15). Importantly, even if patients received prior CD20-targeted therapy, i.e., rituximab and/or obinutuzumab, epcoritamab mediated effective cytotoxicity (median 74%; range 42–95%; *n* = 10) (Fig. 1A, B). CD20 expression levels on tumor cells in CD20-naïve patient samples did not correlate with epcoritamab-dependent cytotoxicity (Fig. 1C). The relation between target expression and cytotoxicity was not explored for R/R patient samples, as target expression could not be reliably quantified due to potential residual membrane-bound CD20 mAbs (rituximab or obinutuzumab). In CD20 mAb-exposed patient samples, sensitivity to epcoritamab correlated with the elapsed time between the last CD20-therapy and the LN biopsy (range: 2 weeks–5 years; *r* = 0.78; *p = 0.03; Fig. 1D). However, most relevant for clinical application is the observation that epcoritamab could induce 42% cytotoxicity in an FL sample that was collected only two weeks after the last anti-CD20 therapy.

### T-cell activation by epcoritamab does not correlate strongly with cytotoxicity of B-NHL cells

**Fig. 2:**
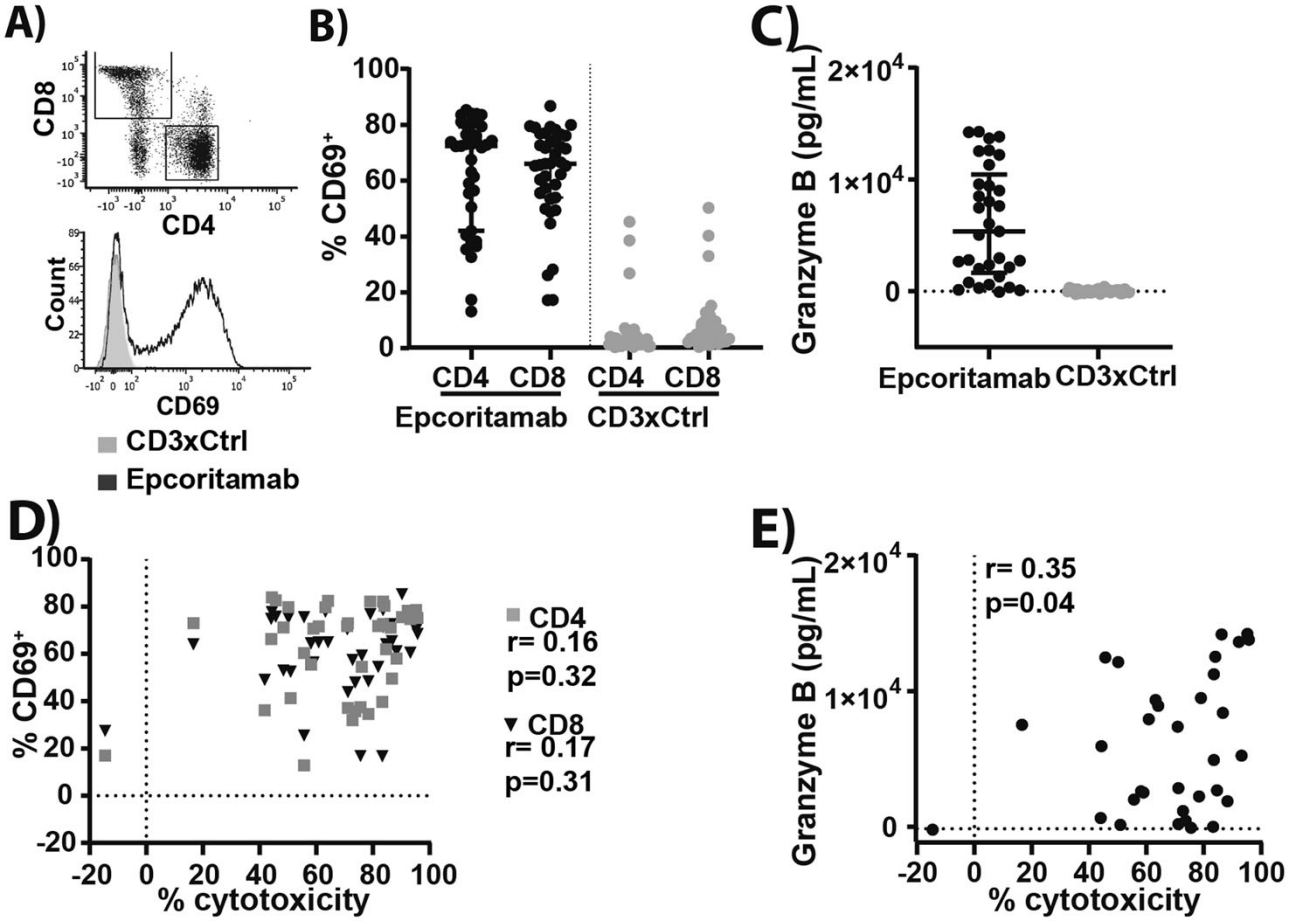
T-cell activation by epcoritamab does not correlate strongly with cytotoxic activity. **A** Gating of CD4^+^ and CD8^+^ cells and example of a flow histogram for CD69 positivity and **B** percentages of CD69-positive CD4^+^ and CD8^+^ allogeneic T-cells after treatment of B-NHL samples with epcoritamab and CD3xCtrl (30 ng/mL) (median ± interquartile range). **C** Granzyme B levels (pg/mL) in supernatants of coculture of B-NHL sample and allogeneic T-cells stimulated with epcoritamab and CD3xCtrl (30 ng/mL) (median ± interquartile range). **D**, **E** Correlation between percentage CD69-positive (CD4^+^ and CD8^+^) allogeneic T-cells (**D**) or granzyme B release (pg/mL) (**E**), and cytotoxicity induced by epcoritamab (30 ng/mL) (Spearman’s; ns and *r* = 0.35; *p = 0.04, respectively). All samples from different B-NHL subtypes and from ND and RR are plotted together.

While epcoritamab consistently mediated cytotoxicity in B-NHL cells, we observed considerable heterogeneity in epcoritamab-induced T-cell activation, as assessed by the induction of CD69 (%) on CD4^+^ and CD8^+^ T-cells, and in total granzyme B release (Fig. 2A–C). Although we observed a significant correlation between T-cell activation and granzyme B production (Supplementary Fig. 1), there was no significant correlation between T-cell activation and killing of malignant cells. A weak but significant correlation was observed between granzyme B levels and epcoritamab-dependent cytotoxicity (*r* = 0.35, *p = 0.04) (Fig. 2D, E). Remarkably, although in some samples very high levels of granzyme B release were observed, the cytotoxicity was relatively low. This suggests that tumor cell-intrinsic resistance mechanisms may play a role in sensitivity to epcoritamab-dependent cytotoxicity. We observed resistance to epcoritamab-dependent cytotoxicity, despite high T-cell activation, for 1 ND DLBCL sample.

### T-cell activation in presence of epcoritamab shows an inverse association with tumor cell HVEM expression levels

**Fig. 3:**
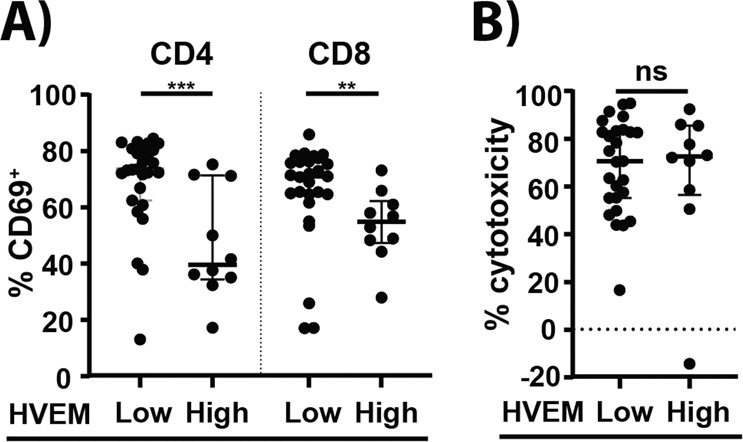
Lower T-cell activation by epcoritamab in presence of tumor cells with high expression of the immune regulatory molecule HVEM. **A** CD69-positive CD4^+^ and CD8^+^ allogeneic T-cells after treatment of B-NHL samples with epcoritamab (30 ng/mL) and CD3xCtrl (30 ng/mL) in samples with low or high tumor HVEM expression levels (Mann–Whitney *U*-test; ***p < 0.001, **p < 0.01). **B** Epcoritamab-dependent cytotoxicity (30 ng/mL) in B-NHL samples with low and high tumor cell HVEM expression (Mann–Whitney *U*-test; ns). HVEM expression on healthy donor B-cells was used as cut-off value to stratify between low and high expression. Data are shown as median ±interquartile range. All samples from different B-NHL subtypes and from ND and RR are plotted together.

To understand the differences in capacity of B-NHL cells to activate healthy donor T-cells in the presence of epcoritamab, we investigated the association between B-NHL subtype, treatment status, and CD20 expression level and between expression of ligands of the immune checkpoint molecules PD-1, LAG3, and BTLA: PD-L1, HLA-DR, and HVEM (tumor necrosis factor receptor superfamily member [TNFRSF]14), with allogeneic T-cell activation. No association between T-cell activation and B-NHL subtype or treatment status was observed (Supplementary Fig. 2), nor between T-cell activation and tumor expression of CD20, PD-L1, or HLA-DR (Supplementary Fig. 3). Activation of allogeneic CD4^+^ and CD8^+^ T-cells was highest in patients that showed low expression of HVEM (i.e., comparable to healthy donor B cells) on B-NHL cells (Fig. 3A). T cell activation was significantly lower in patients that showed higher tumor HVEM expression, potentially indicating tumor cell-mediated immune suppression in these samples. However, no relation between HVEM expression and epcoritamab-dependent cytotoxicity was observed (Fig. 3B).

### B-NHL LN T-cells induce epcoritamab-dependent cytotoxic activity

**Fig. 4:**
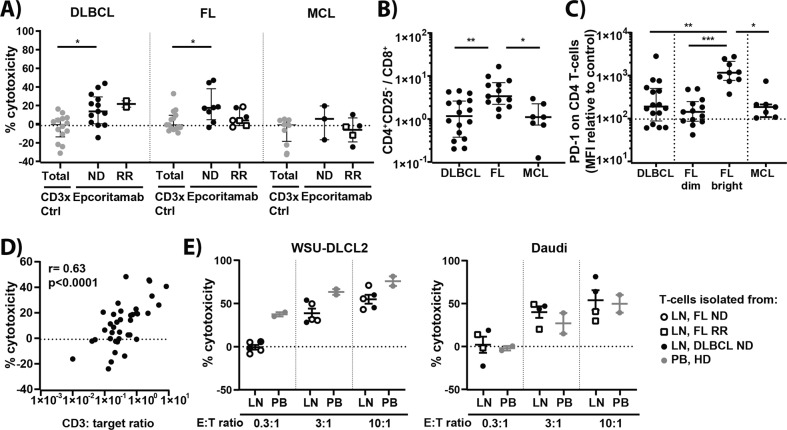
The intrinsic capacity of T-cells from B-NHL LN samples to induce cytotoxicity is comparable to that of healthy donor T-cells. **A** Percentages cytotoxicity mediated by epcoritamab and CD3xCtrl (30 ng/mL) without addition of PBMCs in ND and RR DLBCL (*n* = 15), FL (*n* = 15), and MCL (*n* = 8) samples (median ± interquartile range), including patients who relapsed from or were refractory to CD20 therapy ( and , respectively). Statistical analysis was performed with Kruskal–Wallis and Dunn’s multiple comparisons test to compare epcoritamab with CD3xCtrl (*p < 0.05) and to compare epcoritamab-dependent cytotoxicity (subtracted with CD3xCTRL values) between subtypes (ns). **B** Ratio of CD4^+^ T helper cells (CD4^+^CD25^−^) to CD8^+^ T-cells in DLBCL, FL, and MCL samples (Kruskal–Wallis with Dunn’s multiple comparisons test; *p < 0.05, **p ≤ 0.01) (median ± interquartile range). **C** PD-1 expression (MFI values normalized to expression on T-cells of internal healthy donor control) on CD4^+^ T-cells of DLBCL, FL, including PD-1 bright and dim populations, and MCL (Kruskal–Wallis with Dunn’s multiple comparisons test; *p < 0.05, **p < 0.01, ***p < 0.001) (median ± interquartile range). **D** Spearman’s correlation between CD3:Target ratio and cytotoxicity mediated by epcoritamab (30 ng/mL) (*r* = 0.63; ****p < 0.0001). **E** Lymph node T-cells of B-NHL patients isolated by magnetic-activated cell-sorting (MACS) showed similar percentages B-cell lymphoma cell line (WSU-DLCL2 and Daudi, left to right) cytotoxicity as T-cells isolated from healthy donor PB (mean ± SEM) (Mann–Whitney *U*-test; ns).

After determining the intrinsic sensitivity of B-NHL cells to epcoritamab-dependent cytotoxicity, we assessed the antitumor activity of epcoritamab in B-NHL samples in the autologous setting, without addition of PBMCs, thus exclusively depending on lymph node-residing autologous T-cells. In this setting, we observed low to moderate levels of cytotoxicity in DLBCL (median 20%; range 0–46%; *n* = 15), FL (median 17%; range 0–46%; *n* = 15) and MCL (median 0%; range 0–11%; *n* = 8) (Fig. 4A), which were overall lower response levels than in the allogeneic setting. To understand the lower levels of cytotoxicity in the autologous setting, we analyzed the phenotype and frequency of T-cells derived from B-NHL patient samples. The samples from FL patients contained significantly higher CD4:CD8 T-cell ratios compared to samples from DLBCL and MCL patients (Fig. 4B), but not specifically higher frequencies of CD4^+^ regulatory T-cells (Supplementary Fig. 4). Both PD-1 bright and dim CD4^+^ T-cell populations were observed in FL samples (Fig. 4C), consistent with the reported presence of PD-1 bright follicular CD4^+^ T-helper cells^15^. Nonetheless, the phenotypic differences among B-NHL subtypes showed no association with the observed cytotoxicity levels. However, we observed a strong correlation between the frequency of total CD3^+^ T-cells present in the single cell suspensions and epcoritamab-dependent cytotoxicity (*r* = 0.63; ****p < 0.0001) (Fig. 4D). This correlation was less profound, but still significant, in the case of CD3^+^CD8^+^ cytotoxic T-cells and CD3^+^CD4^+^ T-helper cells (*r* = 0.46; **p = 0.004 and *r* = 0.57; ***p = 0.0002, respectively) (Supplementary Fig. 5). To confirm that the T-cell content of single cell suspensions used in experiments depicted in Fig. 4A–D was representative for the intact LN biopsies, we performed multiplexed immunofluorescence (mIF) analyses. Here, similar numbers of CD4^+^ and CD8^+^ T-cells were found in matched FFPE LN biopsies (*n* = 23) (Supplementary Fig. 6).

We next studied whether CD3^+^ T-cells in the B-NHL microenvironment showed any deficiencies in their capacity to induce epcoritamab-dependent kill of B-NHL cells, by comparing MACS sorted T-cells from four DLBCL, five FL LN biopsies and four healthy donor PBMCs. T-cells from B-NHL LN biopsies mediated comparable levels of epcoritamab-dependent cytotoxicity as T cells-cell from healthy individuals against two different B-NHL cell lines (Daudi and WSU-DLCL2) at three different E:T ratios (Fig. 4E). These results indicated the sufficient capacity of T cells derived from B-NHL biopsies to mediate epcoritamab-dependent cytotoxicity.

### B-NHL peripheral blood T-cells induce epcoritamab-dependent cytotoxicity

**Fig. 5:**
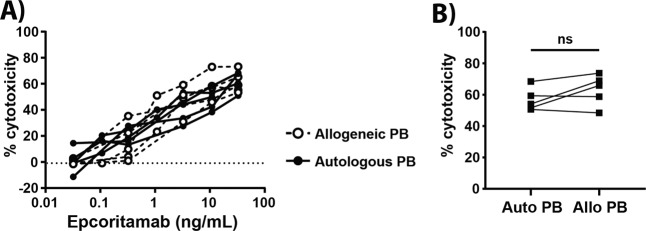
The intrinsic capacity of T-cells from peripheral blood of B-NHL patients to induce cytotoxicity is comparable to that of healthy donor T-cells. **A** Dose-response of and **B** maximal cytotoxicity induced by epcoritamab in B-NHL cells in the presence of allogeneic healthy donor PBMCs or autologous B-NHL patient PBMCs in a 10:1 E:T ratio (*n* = 5; Wilcoxon matched-pairs signed rank test; ns).

The presence of low T-cell numbers in the tumor microenvironment of B-NHL patients suggested that the efficacy of epcoritamab in vivo might depend on infiltration of T-cells from peripheral blood. We therefore analyzed whether autologous peripheral blood T-cells from B-NHL patients were capable of mediating epcoritamab-dependent cytotoxicity. In all available samples (*n* = 5), patient-derived PBMCs showed comparable capacity to induce epcoritamab-dependent cytotoxicity as healthy donor PBMCs (median of 54% vs. 66% cytotoxicity (*p* = ns), respectively) (Fig. 5A, B). All together, these results indicate that there were no apparent intrinsic dysfunctions in peripheral blood and LN-derived T-cells from B-NHL patients which could negatively affect the efficacy of epcoritamab in the clinical setting.

## Discussion

In this study, we evaluated the preclinical efficacy of epcoritamab, a novel full-length IgG1 CD3 bsAb that triggers T-cell-mediated cytotoxicity of CD20-positive tumor cells^7^. First, using T-cells from a single healthy donor as effector cells, we explored the intrinsic sensitivity of tumor cells from different B-NHL subtypes to epcoritamab and their capacity to activate T-cells. We demonstrate that epcoritamab mediated high levels of cytotoxicity against tumor cells from patients with B-NHL (DLBCL, FL, and MCL). We observed some heterogeneity in the level of epcoritamab-dependent cytotoxicity, which could not be explained by CD20 expression levels or B-NHL subtype. Importantly, epcoritamab was equally effective in samples from ND patients and from RR patients, including patients who relapsed from or were refractory to prior anti-CD20-therapy. In RR patients there was a weak but significant correlation between epcoritamab-dependent cytotoxicity and the time elapsed, since the last anti-CD20-treatment was given to the patients. It is known that anti-CD20 mAbs can be present in the circulation for long periods of time, and therefore could compete with epcoritamab for antigen binding^16,17^. However, largely ruling out this possibility, it has recently been shown that the potency of epcoritamab in vivo is not affected by a large excess of rituximab in the circulation^7^. Another explanation for the observed correlation could be the CD20 mAb induced CD20 down-modulation, although CD20 expression after CD20 mAb treatment was reported to be fairly stable in the majority of lymphoma patients^18–20^. CD20 expression levels could however not be reliably assessed in this study, due to competition of the detection antibody with potential residual membrane-bound CD20 mAb. Nevertheless, we still observed a substantial response of 42% to epcoritamab in a sample that was collected only 2 weeks after last exposure to CD20-treatment, indicating that epcoritamab can be effective even if it is administered shortly after discontinuing treatment with a CD20 mAb.

Another interesting observation in our study is the heterogeneity in the level of activation in allogeneic T cells after exposure to B-NHL in the presence of epcoritamab. As one single donor was used in the experiments, differences may not be attributed to intrinsic differences in the allogeneic T cell population. Conversely, this approach did allow to investigate intrinsic differences in B-NHL studied. PD-L1 or HLA-DR expression in B-NHL did not show a correlation with T-cell activation. Interestingly, we observed an inverse association of T-cell activation levels with expression of the immune checkpoint molecule HVEM on tumor cells. Hence, this observation indicates that immune checkpoint pathways may play a role in modulation of T-cell functionality. Furthermore, the lack of a strong correlation between T-cell activation and granzyme B release with epcoritamab-dependent cytotoxicity indicates the involvement of yet unknown intrinsic resistance mechanisms in B-NHL cells against the cytotoxic activity of T-cells. Remarkably, these resistance mechanisms are especially visible when CD20 expression levels are relatively lower, which suggests that higher target expression can overcome these resistance mechanisms. These observations warrant further investigation. It would be interesting to investigate the possibility of increasing the level of target expression or T-cell activation to potentially overcome the intrinsic resistance.

In this study we also performed a detailed analysis of the B-NHL patients’ own T-cells to gain deeper insight into their capacity to mediate epcoritamab-dependent cytotoxicity. Without the addition of PBMCs, we observed that epcoritamab-dependent cytotoxicity correlated significantly with the local frequency of CD3^+^ T-cells in the LN. T-cells residing in LN showed no functional deficiencies, as evidenced by the fact that they were capable of inducing sufficient levels of epcoritamab-dependent cytotoxicity especially at effector to target ratios higher than 3:1. In addition, autologous peripheral blood T-cells from B-NHL patients as well as from healthy individuals induced comparable levels of tumor cell kill in the presence of epcoritamab. Thus, T-cells present in the LN tumor microenvironment, as well as T-cells isolated from the peripheral blood of B-NHL patients possess adequate capacity to mediate epcoritamab-dependent cytotoxicity. Our results suggest that the recruitment of sufficient peripheral blood T cells into the LN will be required for optimal clinical efficacy of epcoritamab. Alternatively, local expansion of tumor-resident T cells may contribute to antitumor activity in patients.

In conclusion, we have demonstrated induction of effective T-cell-mediated cytotoxicity by epcoritamab against primary B-NHL cells, and thereby provide preclinical proof-of-concept for the treatment of B-NHL patients with epcoritamab, including patients who relapsed after prior CD20-mAb containing treatment. Moreover, we show that peripheral blood or tumor-resident T cells from B-NHL patients show excellent capacity to induce T-cell-mediated cytotoxicity in presence of epcoritamab. These data support the single-agent activity of epcoritamab demonstrated in the ongoing first in human trial (NCT03625037^14^) in patients with relapsed, progressive, or refractory B-cell lymphoma, and warrant further investigation of specific biomarkers that may be correlated with response to epcoritamab.

## Supplementary information

Supplemental material and figures
