# Supplementary material for: Epcoritamab induces potent anti-tumor activity against malignant B-cells from patients with DLBCL, FL and MCL, irrespective of prior CD20 monoclonal antibody treatment
Source: Blood Cancer J. 2021 Feb 18;11(2):38. doi: 10.1038/s41408-021-00430-6 (PMC7892878; doi:10.1038/s41408-021-00430-6)
Supplement: Supplementary file 1 — Supplemental material and figures [file 41408_2021_430_MOESM1_ESM.docx]

**Epcoritamab induces potent anti-tumor activity against malignant B-cells from patients with DLBCL, FL and MCL, irrespective of prior CD20 monoclonal antibody treatment**

**Supplemental material and methods**

**Lymph node (LN) suspension cells**

LN biopsies were mechanically disrupted into small fragments and cultured in RPMI 1640 + 10% fetal bovine serum (Invitrogen, Carlsbad, CA, USA) in a fully humidified incubator at 37˚C, 5% CO_2_ for 16 hours. The supernatant was filtered using a 70 µM Easy Strainer (Greiner Bio-one, Alphen aan den Rijn, the Netherlands) to collect the malignant B-cells, T-cells and other cells diffused out of the biopsy fragments.

**Normalization of flow-based phenotyping**

Median fluorescence intensity (MFI) values were normalized to expression levels on B – and T-cells of a single healthy donor whose PBMCs were measured parallel to each B-NHL sample, to minimize inter-experimental variations. The expression levels on B- and T-cells of this healthy donor were also used as cut-off value to stratify between low and high expression levels on B-NHL cells. PD-1 expression was defined as dim and bright if two clear separate populations were observed.

**Flow cytometry-based cytotoxicity assays**

In the flow cytometry-based cytotoxicity assays violet tracer-negative allogeneic CD3^+^ donor T-cells or violet tracer-positive CD3^+^ patient T-cells were examined for expression of the T-cell activation marker CD69.

All antibodies and markers used in flow cytometry-based B- and T-cell phenotyping and cytotoxicity assays are depicted in Table 1.

Table 1 Antibodies used in flow cytometry for tumor and T-cell phenotyping and cytotoxicity assays

| **Target** | **Label** | **Assay** | **Company** |
| --- | --- | --- | --- |
| CD20 | V450 | Tumor phenotyping | BD Horizon |
| CD19 | PC7 | Tumor phenotyping | Beckman Coulter |
| CD45 | Krome Orange | Tumor phenotyping Cytotoxicity | Beckman Coulter |
| Kappa | PE | Tumor phenotyping | DAKO |
| Lambda | FITC | Tumor phenotyping | Emelca Bioscience |
| CD274 | BV786 | Tumor phenotyping | BD |
| HLA-DR | APC-H7 | Tumor phenotyping | BD |
| CD270 | BV711 | Tumor phenotyping | BD |
| CD5 | APC | Tumor phenotyping | BD |
| CD10 | APC | Tumor phenotyping | BioLegend |
| Live/dead | BUV455 | Tumor phenotyping | Invitrogen |
| CD3 | BUV395 | T-cell phenotyping | BD |
| CD4 | APC-H7 | T-cell phenotyping Cytotoxicity | BD |
| CD8 | PC7 | T-cell phenotyping | BD |
| CD279 | BV605 | T-cell phenotyping | BioLegend |
| CD25 | FITC | T-cell phenotyping | BioLegend |
| CD366 | PE | T-cell phenotyping | BD |
| CD272 | V450 | T-cell phenotyping | BD |
| CD127 | PE-CF594 | T-cell phenotyping | BD |
| CD223 | APC | T-cell phenotyping | BD |
| CD197 | BV786 | T-cell phenotyping | BD |
| CD45RA | AF700 | T-cell phenotyping | BD |
| 7-AAD |  | T-cell phenotyping Cytotoxicity | BD |
| CD2 | BV786 | Cytotoxicity | BD |
| CD8 | APC R700 | Cytotoxicity | BD |
| CD19 | BUV737 | Cytotoxicity | BD |
| CD25 | PE | Cytotoxicity | DAKO |
| CD69 | FITC | Cytotoxicity | BioLegend |
| *CD, cluster of differentiation; V, violet; PC7,* *phycoerythrin cyanin 7; PE, phycoerythrin; FITC, fluorescein isothiocyanate; BV, briljant violet; HLA, human leukocyte antigen; APC, allophycocyanin; H7, cyanine 7 (Cy7); BUV, briljant ultraviolet; AF, alexa fluor; 7-AAD, 7-amino-actinomycin D* | | | |

**Multiplexed immunofluorescence (mIF)**

Slides were deparaffinized and rehydrated, followed by a blocking step for endogenous peroxidase using 0.3% H_2_O_2_/methanol and fixation with 10% neutral buffered formalin (Leica Biosystems, Nußloch, Germany). Slides were washed in Milli-Q water and 0.05% Tween20 in 1x Tris-Buffered Saline (TBS-T). Antigen retrieval was done by placing the slides in 0.05% ProClin300 (Sigma-Aldrich, Saint Louis, MO, USA)/Tris–EDTA buffer pH 9.0 in a microwave at 100% power until boiling point, followed by 15 min at 30% power. Slides were cooled in Milli-Q water, washed in 1x TBS-T and blocked with Antibody Diluent (Agilent Technologies, Santa Clara, CA, USA). The slides were then incubated with primary antibody diluted in Normal Antibody Diluent, followed by incubation with the broad spectrum horseradish peroxidase (HRP) from the SuperPicture Polymer Detection Kit (Life Technologies, Carlsbad, CA, USA). Next, the slides were incubated with Opal TSA fluorochromes (Akoya biosciences, Menlo Park, CA, USA) diluted in amplification buffer (Akoya biosciences). The primary and secondary antibody complex was stripped by microwave treatment with 0.05% ProClin300/Tris–EDTA buffer at pH 9.0. The combination of primary antibody and fluorescent dyes is indicated in table 2, in order of staining. Finally, DAPI working solution (Akoya biosciences) was applied and the slides were mounted with Prolong Diamond Anti-fade mounting medium (#P36965; Life Technologies).

Table 2 Antibodies used in multiplexed immunofluorescence

| **Marker** | **Clone (Company)** | **Dilution** | **Opal fluorophore** |
| --- | --- | --- | --- |
| PD-1 | EH33 (Cell signaling) | 1:500 | Opal520 |
| CD8 | C8/144B (Agilent) | 1:1000 | Opal620 |
| CD3 | CD3 (Agilent) | 1:500 | Opal690 |
| PD-L1 | 22C3 (Agilent) | 1:100 | Opal650 |
| PAX5 | 24/PAX-5 (BD Biosciences) | 1:100 | Opal570 |
| CD163 | 10D6 (Novocastra) | 1:500 | Opal540 |
| *PD-1, programmed cell death protein 1; PD-L1, programmed death-ligand 1; PAX5, paired box 5* | | | |

**Image acquisition and quantification**

Stained slides were scanned using the Vectra Polaris Automated Quantitative Pathology Imaging System (Akoya biosciences). For each sample, representative tumor regions were selected and six 2x2 fields of view were acquired at 40x resolution. After image capture, the images were spectrally unmixed and analyzed, using supervised machine learning algorithms within Inform 2.4.1. (Akoya biosciences). In brief, individual cells were located and segmented to be able to perform analysis on a per-cell basis. Cells were then assigned into four different phenotype categories: “T-cell”, “tumor”, “macrophage” or “other”, based on the size of the cells and positivity of markers in the panel. In the training phase, cells representative for each category were manually selected after which the algorithm predicts the phenotype for all remaining cells. To improve accuracy, phenotypes were checked and adjusted when necessary. The training was applied to all six images of each sample. Thresholds for positive staining were determined for all markers. Data was exported and custom R scripts were used to further stratify cells within the phenotype categories, based on thresholds of positivity. Specifically for T-cells: T-helper cells (CD3^+^CD8^-^PD1^-^ and CD3^+^CD8^-^PD1^+^) and cytotoxic T-cells (CD3^+^CD8^+^PD1^-^ and CD3^+^CD8^+^PD1^+^). For tumor, macrophages and other, cells were further stratified into PD-L1 negative and PD-L1 positive. Phenotypes were then summarized per sample using the phenoptr R package (1).

**Supplemental figures**

**
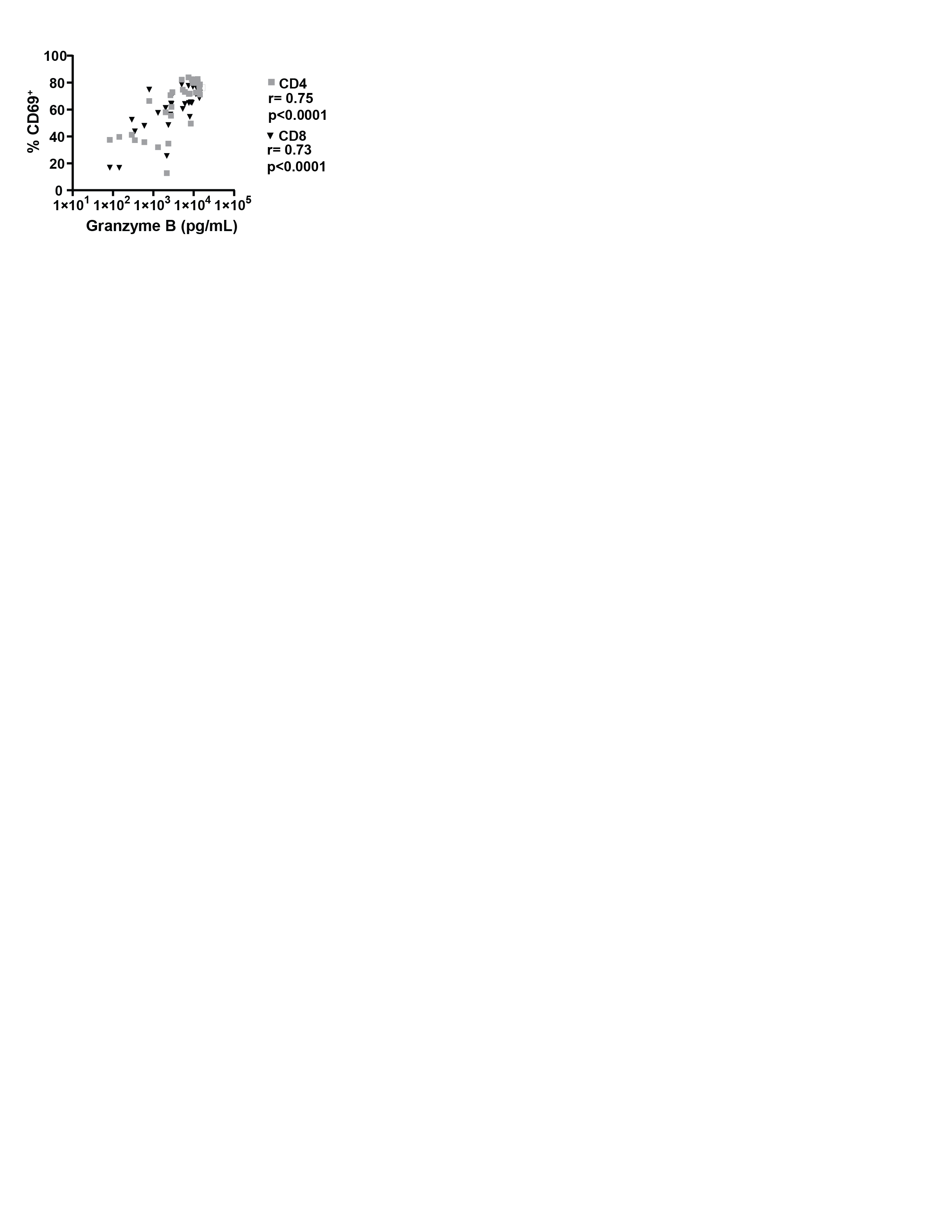
**

**Supplemental figure 1. T-cell activation correlates with release of granzyme B.** Spearman’s correlation of epcoritamab-mediated (30 ng/mL) CD69 positivity on CD4^+^ and CD8^+^ allogeneic T-cells and granzyme B release (pg/mL) by these T-cells in the presence of B-NHL cells (****p<0.0001).

**
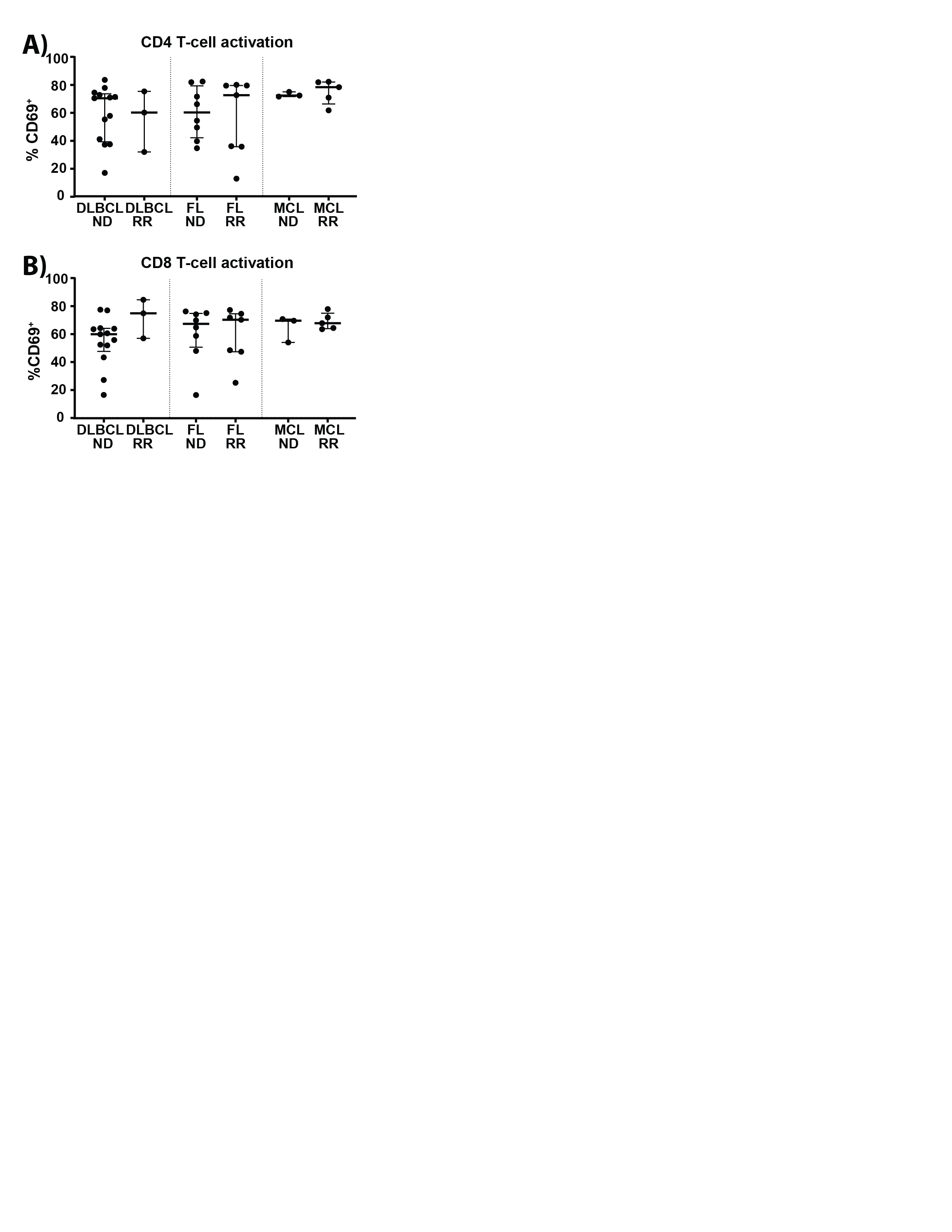
**

**Supplemental figure 2. Epcoritamab induces similar levels of allogeneic T-cell activation in the presence of different subtypes of ND and RR B-NHL. (A,B)** CD69 positive CD4^+^ and CD8^+^ allogeneic T-cells upon epcoritamab treatment (30 ng/mL) in the presence of B-NHL samples for different subtypes and for ND and RR. Data are shown as median ± interquartile range.


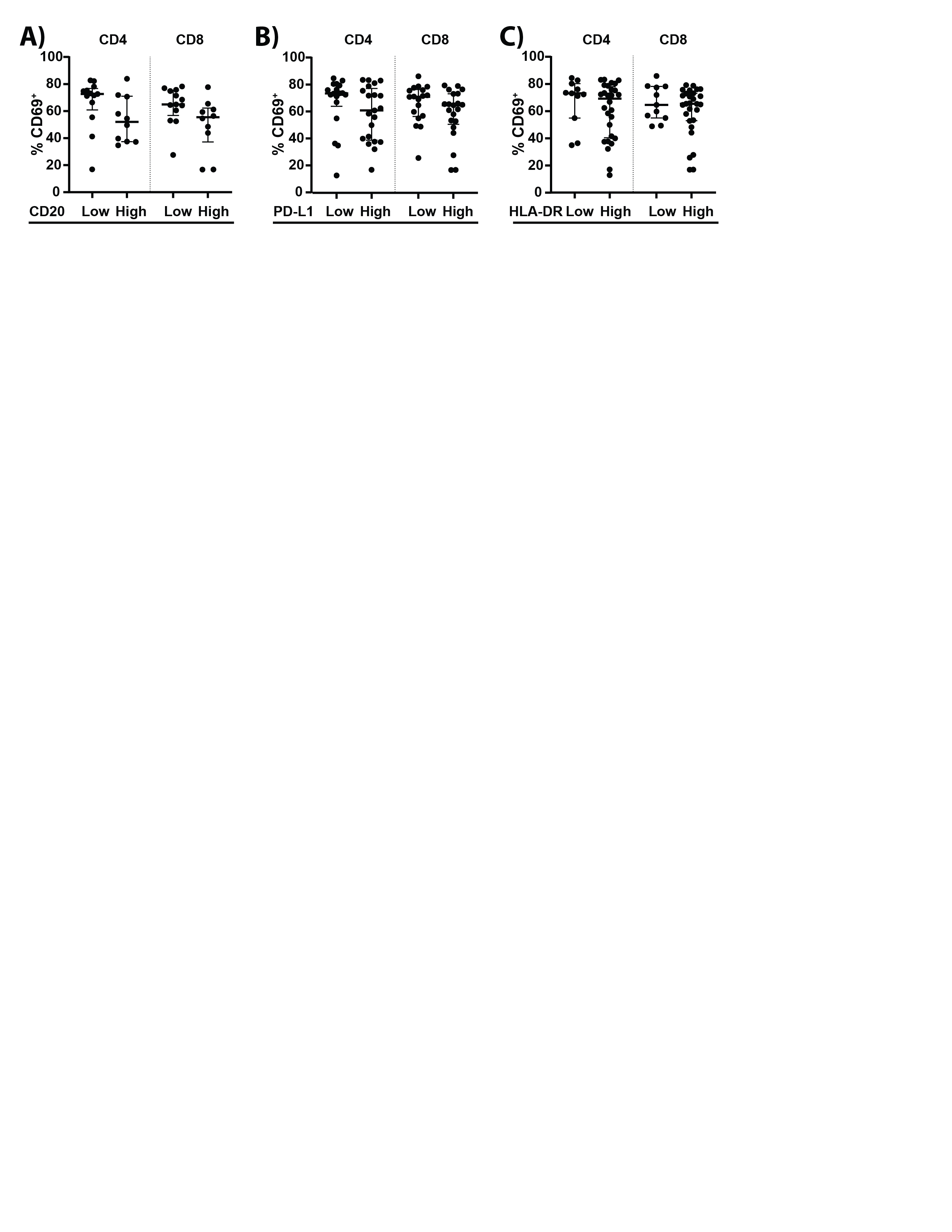


**Supplemental figure 3.** **(A-C)** CD69-positive allogeneic CD4^+^ and CD8^+^ T-cells induced by epcoritamab (30 ng/mL) in B-NHL samples with low and high tumor cell CD20 (A), PD-L1 (B) and HLA-DR expression (C) (Mann-Whitney U-test; ns). Median CD20 expression (antibody molecules per cell), defined on samples of ND patients, was used to stratify between low and high CD20 expressing ND samples. PD-L1 and HLA-DR expression on healthy donor B-cells was used as cut-off value to stratify between low and high PD-L1 and HLA-DR expression. Data are shown as median ± interquartile range.


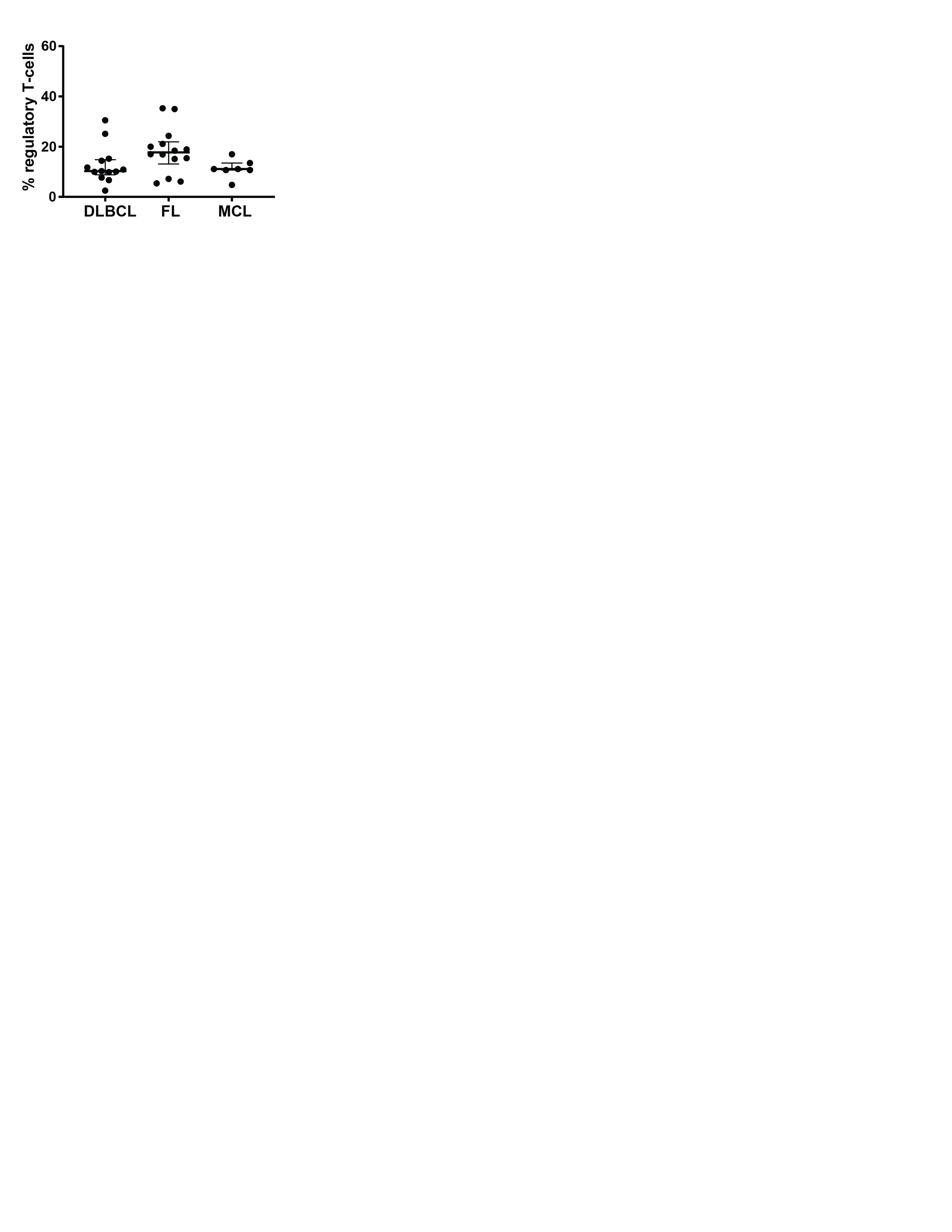


**Supplemental figure 4. Comparable frequencies of regulatory T-cells in different B-NHL subtypes.** Percentage of CD25^+^CD127^low/-^ regulatory T-cells in the CD4^+^ T-cell population in DLBCL, FL and MCL samples. Data are shown as median ± interquartile range (Kruskal-Wallis with Dunn’s multiple comparisons test; ns).

**
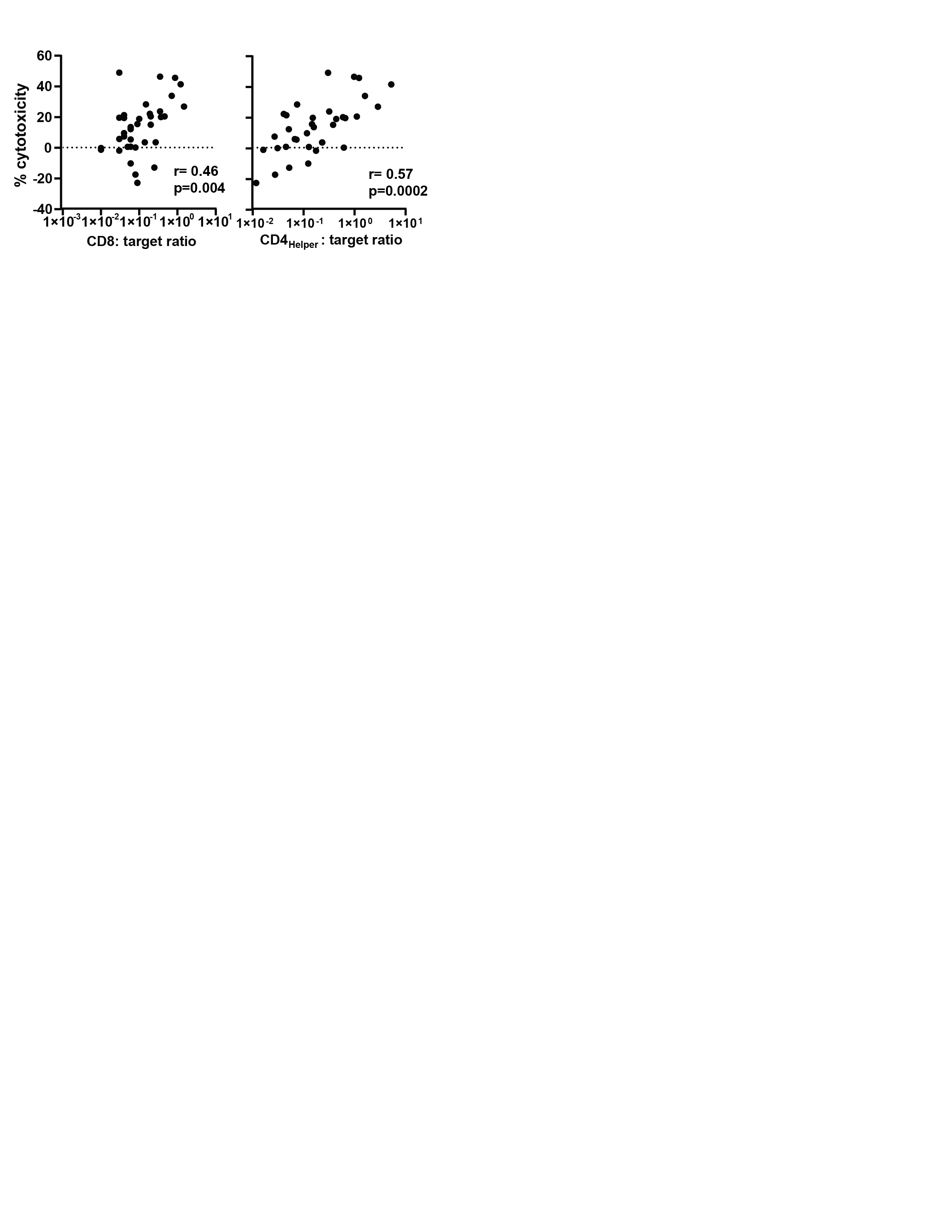
**

**Supplemental figure 5**. **Epcoritamab-dependent cytotoxicity correlates with both CD4:Target and CD8:Target ratios.** Spearman’s correlation of epcoritamab-dependent cytotoxicity (30 ng/mL) in B-NHL samples with the ratios of CD8^+^ T-cells to target cell (left) and CD4^+^ T helper cells to target cell (right) (r=0.46; **p=0.004 and r=0.57; ***p=0.0002, respectively).

**
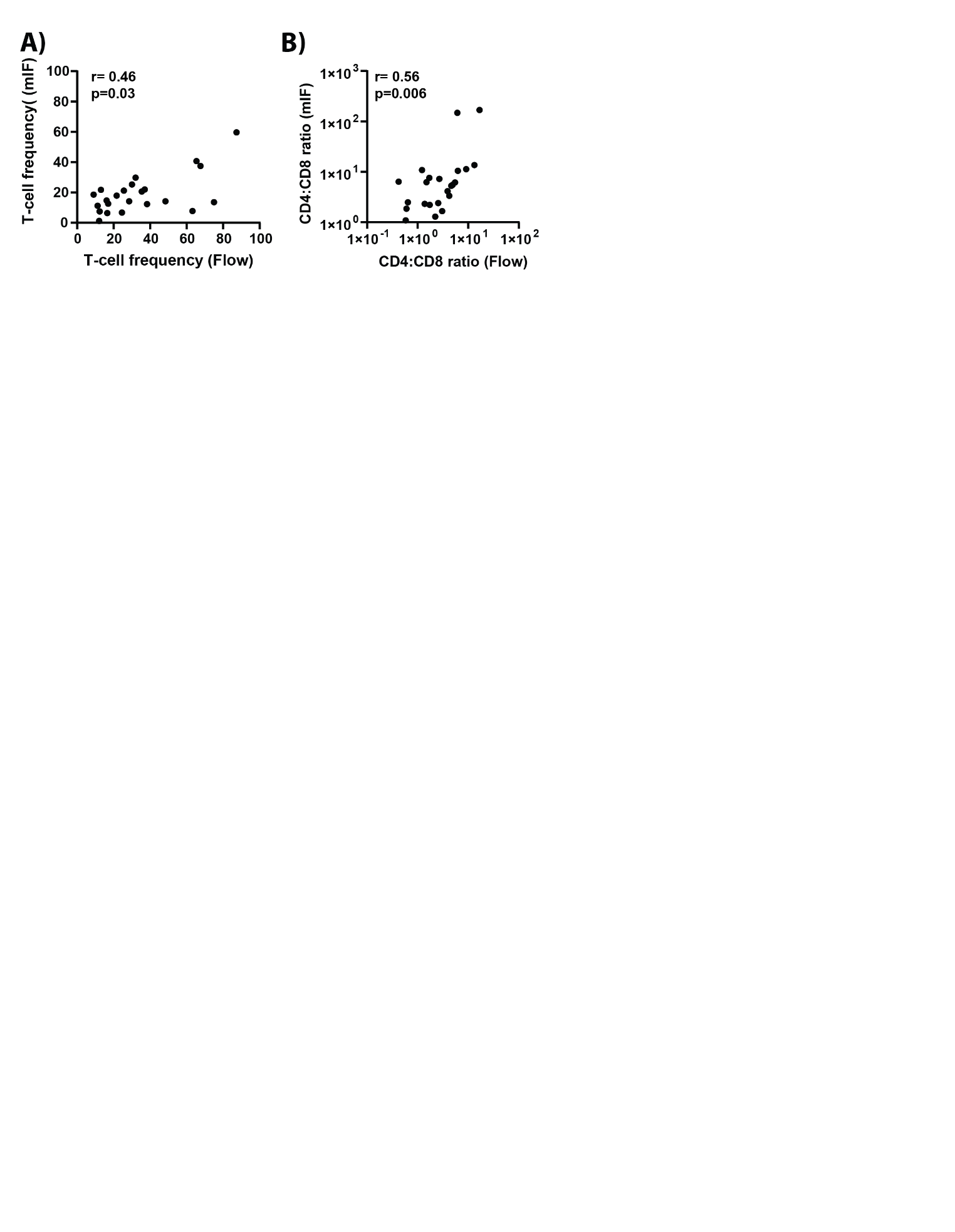
Supplemental figure 6.** **T-cell content of single cell suspensions is representative for intact LN biopsies.** Spearman’s correlation of flow cytometry-based T-cell frequencies in lymph node suspension cells with multiplexed immunofluorescence (mIF)-based T-cell frequencies in lymph node biopsies. **(A)** CD3^+^ T-cell frequency (r=0.46, *p=0.03) and **(B)** CD4:CD8 T-cell ratio (r=0.56, **p=0.006).

**References**

1. Johnson KS. Phenoptr:inForm Helper Functions. R package version 0.2.5. 2020.
